# Teleology of immune system response to sepsis – failure due to dysregulation or adaptive response that sometimes fails?

**DOI:** 10.3389/fimmu.2026.1845463

**Published:** 2026-05-28

**Authors:** Krzysztof Laudanski

**Affiliations:** Department of Anesthesiology and Perioperative Care, Mayo Clinic, Rochester, MN, United States

**Keywords:** Critical grade insult, septic shock, immune response, teleology, homeostasis, allostasis, long-term outcomes

## Abstract

Critical-grade insults (CGIs), such as sepsis, pose extreme challenges to host homeostasis. Although unfavorable immune responses in these conditions are commonly described as dysregulated, such perturbations are often defined retrospectively based on clinical outcomes rather than on the underlying immune processes. Here, I propose that, particularly in its early phase, the immune system response to sepsis reflects a high-stakes host response aimed at rapid control of the insult, albeit one that carries substantial risk to the host. If the inciting challenge is effectively contained, the immune response may resolve and transition toward tissue repair and recovery. Conversely, failure to control the insult may result in host death. In this context, the early immune response can be conceptualized within a “win versus loss” framework, where win is described as the ability to control inciting insult, while loss is host demise. Consequently, the term “dysregulation” may not fully capture the probabilistic feature of the initial response. Rather than representing a simple failure of control, this response may reflect an aggressive but potentially hazardous strategy that, in some cases, proves unsuccessful. Timely medical interventions, including appropriate antibiotic therapy, source control, or immunological endotype-based strategies, can modify CGI immunological trajectory by limiting insult burden and reducing the duration and intensity of the host response, thereby improving the likelihood of survival. Nevertheless, sustained or excessive early immune activation may disrupt regulatory networks, resulting in inevitable demise in some individuals, while others will exhibit an emergence of maladaptive allostatic states that can impair full recovery.

## Introduction

Sepsis continues to cause significant morbidity and mortality despite intensive efforts to improve care (1, 2). There is a widespread belief that the immune response, designed to control infection, may itself become “overwhelmed” and “dysregulated” during sepsis (3–6). However, the fact that multiple attempts to moderate “dysregulated” immune system activation have proved to have, at best, a moderate, if any, effect on mortality suggests that sepsis is not a problem of immune system “dysregulation” (2, 6). Rather, I propose that the immune system response in sepsis represents the maximal response to contain an existential challenge to the host - yet it will fail in some cases, resulting in inevitable mortality (2). This explains why the only intervention to date that affects mortality is controlling the inciting event through early antibiotic treatment and/or surgical intervention. Novel therapies based on the septic endotypes may further improve mortality, yet elimination of sepsis-related mortality is likely not possible (6, 7). Such interventions can limit the time the immune system has to “bet” survival on a maximal response, even if that response fails (1, 8). In subjects surviving the initial stages of the response, precision medicine may re-regulate the immune response to more optimal levels (7).

## The nature of the immune response

**Figure 1:**
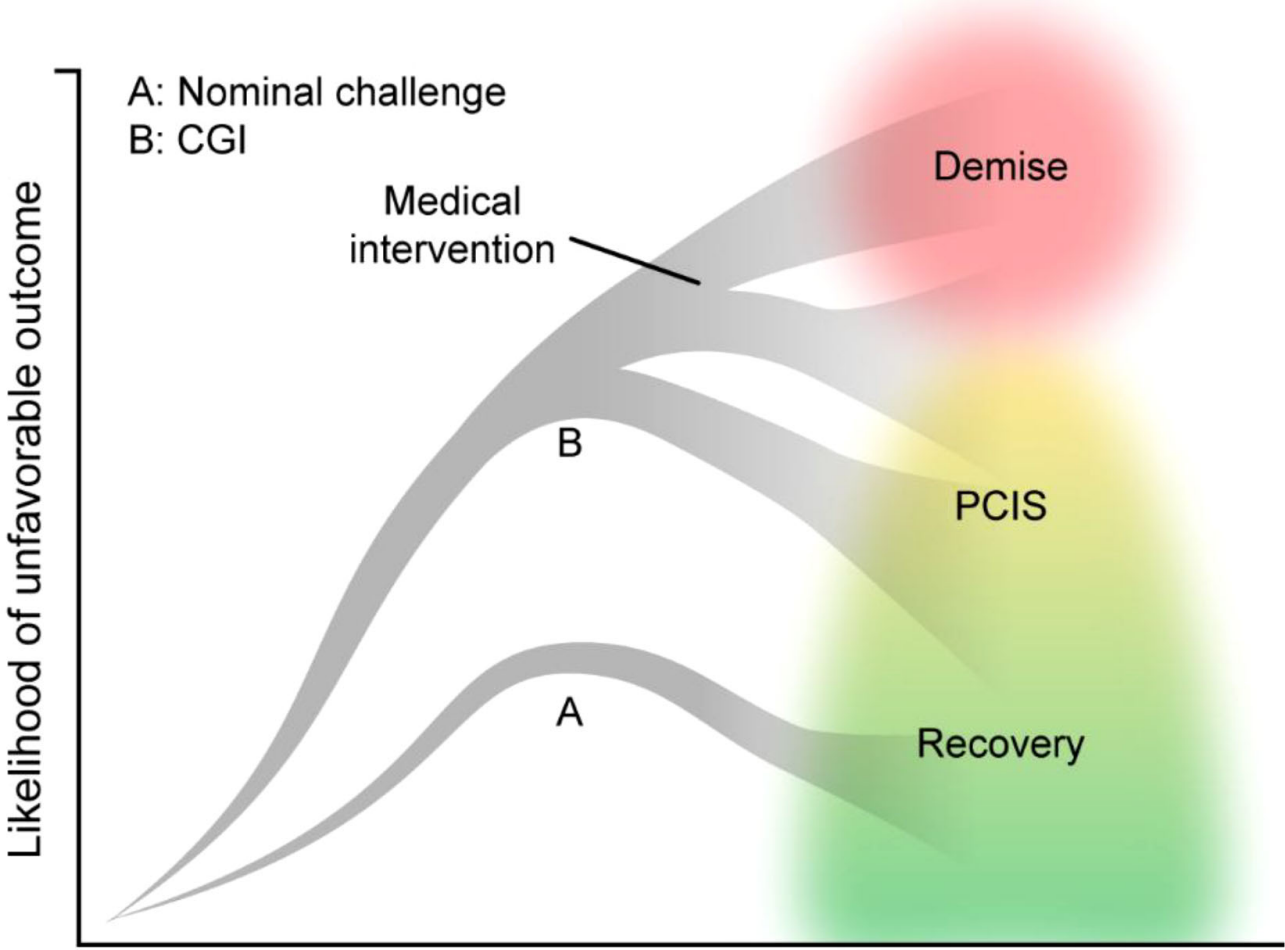
Nominal challenge (marked as A trajectory) results in virtually almost always a recovery of health. In contrast critical grade insult (CGI; marked as trajectory B) triggers a “win versus loss” where maximal immune responses to critical illness may lead to distinct outcomes: preservation or restoration of homeostasis, a transition to a new allostatic state characterized by post-critical illness syndrome (PCIS), or demise. Medical interventions, particularly those targeting the underlying infection, may alter the natural trajectory and improve the likelihood of recovery; however, a subset of individuals may still experience fatal outcomes despite appropriate treatment.

Pathogen-induced activation of the immune system poses the ultimate existential challenge to homeostasis. In effect, the host is making a calculated gamble: generate a maximal, rapid response or succumb to the challenge (*win vs. lose*) (**Figure 1**). This rapid response comes at the expense of specificity, which can lead to “off-target” effects that cause collateral damage to organs. Concomitantly with the initial innate response, an acquired immune response gradually emerges and matures, focusing on eliminating the pathogen, limiting collateral damage, and activating regeneration processes. There are **four** potential outcomes of the immunological responses to sepsis (**Figure 1**): **1)** if the challenge can be contained, the immune system response will subside and shift to a regenerative response; **2)** if the pathogen cannot be controlled, it will lead to the host’s demise, or if the challenge is contained but regulatory refocus regenerative responses are impaired, a new allostatic balance will emerge resulting in **3)** subclinical changes not apparent until next challenge is encountered or **4)** maladaptive allostasis as seen in PCIS, exhaustion, or anergy (4, 9–11). The goal of medical interventions is to favor the first outcome and increase the probability of survival with the re-establishment of homeostasis. Yet even with the early introduction of antibiotics or precision, endotype-driven therapies, an alternative outcome will still occur, albeit at a lower frequency (7, 12, 13).

## A teleological view of sepsis

**Figure 2:**
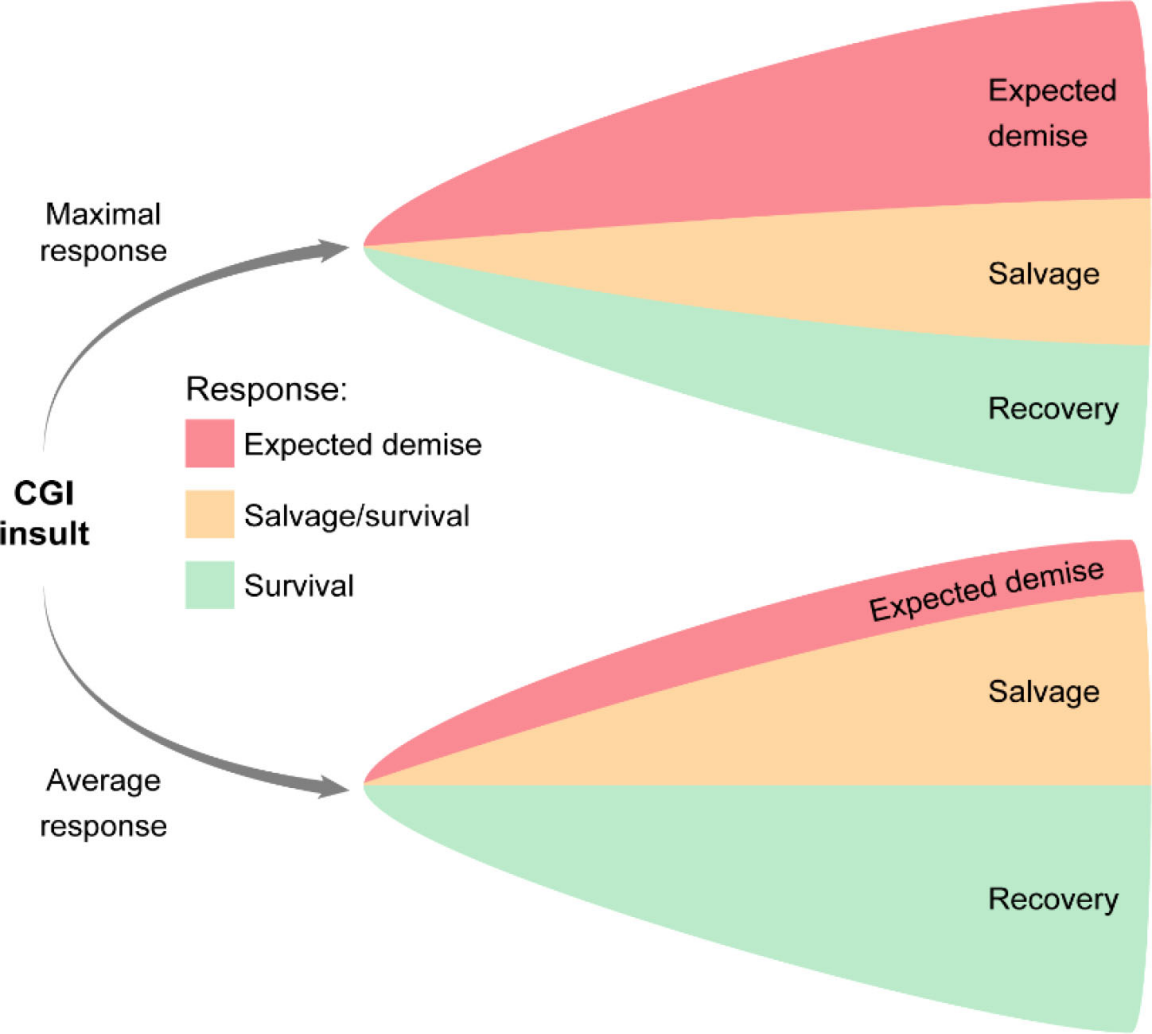
Some unfavorable outcomes following critical-grade insults (CGI), such as sepsis, are likely unavoidable (RED). However, recovery remains a possible outcome (GREEN), particularly with the timely implementation of appropriate therapies, including antibiotics, which increase the probability of survival (YELLOW). Additionally, when the initial insult is below maximal severity, the host immune response may be more proportionate, thereby reducing the likelihood of adverse outcomes.

Given that the sepsis response can contribute to the host’s demise, the question arises whether this is truly an optimal response. From an evolutionary perspective, maladaptive responses are eliminated because they do not confer an ecological advantage (14). Conversely, one could hypothesize that the observed response to sepsis is optimal, or at least evolutionarily advantageous, considering from an evolutionary perspective rather than a detrimental one (14–16). But even the most optimal and maximal response will fail in some instances when the challenge is extreme relative to the coping mechanisms of the host, and death will result. (**Figure 2**) This perspective would view sepsis not as dysregulation of the immune system, even if it fails to contain the CGI challenge. In other words, the response is adequate from a teleological point of view, but the host’s lack of resilience in the face of the challenge leads to the host’s demise. Furthermore, these ancient survival mechanisms optimized for acute infection become maladaptive in modern clinical contexts. This conceptual approach to sepsis has at least two implications:

1. Although sepsis is often viewed as a dysregulated inflammatory response, “dysregulation” is usually defined by clinical outcomes rather than the immunological process itself (10). A teleological view of sepsis would reject such characterizations of the process, even though outcomes (e.g., patient demise) may be unfavorable. In some individuals, the challenge will be too much even with maximal mobilization of the immune system. Thus, the immunological response to the existential threat posed by a pathogen can be viewed as an adaptive response that sometimes fails. A widespread perception of sepsis as a seemingly harmful response to CGI needs to be revised, as such a utilitarian-based judgment of immune reactions to CGI is biased and focuses more on outcomes than on the process.
2. Maximal immunological mobilization is the optimal response, and modulating such a response may be counterproductive in the early stages. To underline this point, multiple clinical trials that attempted to modulate the immune response found a modest, at best, clinically significant impact (1, 8). One could argue that modulating maximal mobilization may be detrimental or ineffective, as a maximal response is precisely what is needed given the pathogen’s dire threat. Reducing the duration of the stressor with antibiotic treatment will tilt the balance of collateral damage toward pathogen control, favoring survival.
3. Post-hyperacute responses to sepsis are much more diverse and set during acute phases. They are most likely to be amenable to precision immunotherapy and clinical intervention to recover health or restore allostasis in others who exhibit maladaptive responses.

## Long-term consequences of sepsis

In the initial 24 to 48 hours, a maximal response requires reprogramming and mobilization of several regulatory networks to be activated in the short term and “win” the struggle against the pathogen (10, 17, 18). If the reprogramming is profound, the transcriptional regulatory network (epigenetic marks, miRNAs, post-translational modifications) can become perturbed, potentially beyond the capacity to recover pre-illness homeostasis (9, 12, 19). So a maximal immunological response may be necessary to cope with a critical event to maximize the host’s ability to contain the insult, yet it will impact the subsequent survivorship if regulatory mechanisms cannot revert to the pre-insult state. Nudiustertian determined changes in the regulatory network and reset homeostatic set points. In such patients, a new balance emerges – allostasis (4). Such patients may manifest persistent smoldering inflammatory syndromes (PCIS), exhaustion, anergy, or chronic critical illness, due to the inability of the immune regulatory networks to restore pre-illness balance (9, 10, 18). In some cases, acquired innate memory will affect the future response even if the clinical impact is not apparent. The optimal time to address long-term immune dysregulation is not very early in the challenge, when regulatory networks are maximally responsive during the acute phase of pathogen clearance, yet are being reprogrammed with long-term impact.

## Summary

Teleological considerations suggest that the immune system’s response to sepsis is evolutionarily optimized to contain threats; otherwise, the host may not survive. Maximum immunological mobilization is often necessary, but in some cases, may remain insufficient for survival. Early medical intervention that alters initial immune activation can be limited in effect or even counterproductive; rather, reducing the severity of the threat may be more effective (20). After containment, the immune system should return to baseline; however, regulatory dysfunction may disrupt this process, affecting both immediate survival and long-term recovery. As the nature and magnitude of immune response observed in other CGI events are relatively consistent across several CGIs, though the triggering insults differ, it remains to be seen if this framework applies to other critical challenges to immunological homeostasis (massive trauma, burn, etc) (12, 21).

## Data Availability

The original contributions presented in the study are included in the article/supplementary material. Further inquiries can be directed to the corresponding author.
